# Not All Weight Loss Is Equal: Towards Muscle‐Preserving Therapies in Obesity

**DOI:** 10.1002/jcsm.70298

**Published:** 2026-04-27

**Authors:** Joseph D. Abraham, Muhammad Shahzeb Khan, Stefan D. Anker

**Affiliations:** ^1^ Heart and Vascular Center, Carl and Edyth Lindner Research Center The Christ Hospital Cincinnati Ohio USA; ^2^ Baylor Scott and White Research Institute Dallas Baylor Scott and White Health Dallas Texas USA; ^3^ Division of Cardiology Baylor Scott and White: The Heart Hospital Plano Plano Texas USA; ^4^ Division of Cardiology Baylor College of Medicine Temple Texas USA; ^5^ Department of Cardiology (CVK) of German, Heart Center Charité, German Centre for Cardiovascular Research (DZHK) Partner Site Charité Universitätsmedizin Berlin Germany

The pharmaceutical therapies targeting obesity have rapidly evolved with the increased study and use of glucagon‐like peptide‐1 (GLP‐1) medications [1, 2]. Treating obesity and overweight, even in the absence of diabetes mellitus, is shown to have beneficial effects on cardiovascular event outcomes [3, 4]. However, an important limitation of this beneficial weight loss is that not all weight loss is equal: 25%–40% of total weight loss may reflect skeletal muscle mass loss or other fat‐free mass [1, 5, 6, 7].

The Phase 2 BELIEVE trial represents a compelling conceptual advance by combining semaglutide with bimagrumab, an activin receptor type II (ActRII) antagonist [8]. Bimagrumab promotes skeletal muscle hypertrophy through modulating myostatin/activin signalling. Via inhibition of the ActRII‐activin receptor‐like kinase 4 (ALK4) pathway, Bimagrumab leads to an anabolic effect in skeletal muscle, and the ActRII‐activin receptor‐like kinase 7 (ALK7) pathway is recognized as contributing to the regulation of obesity [8, 9, 10]. Mechanistically, the muscle weight‐loss associated with GLP‐1 therapies, such as semaglutide, may be mitigated with ActRII antagonism. The BELIEVE trial clinically demonstrated this composition of weight loss in a multicentre, randomized, double‐blind, placebo‐controlled trial.

A total of 507 patients were randomized to nine groups in an equal ratio: placebo, low‐ or high‐dose Bimagrumab, low‐ or high‐dose Semaglutide or combinations of both. The primary endpoint was absolute change from baseline body weight at Week 48. At 48 weeks, weight reduction was observed across all active treatment groups, with greater reductions seen with semaglutide compared to bimagrumab alone. The combination therapy produced the most pronounced effect, achieving significantly more weight loss than either monotherapy and far exceeding placebo. Of greater relevance, secondary endpoints included measures of body composition: absolute and percent change in total body fat and lean mass at Weeks 48 and 72 as measured by dual‐energy X‐ray absorptiometry. Although semaglutide monotherapy was observed to have a reduction in lean mass, bimagrumab preserved or even increased lean mass. Combination therapy attenuated the loss of lean mass seen with semaglutide monotherapy. Furthermore, the proportion of total weight loss from adipose mass was highest in combination therapy. These results show that, with pharmaceutical therapy, it is possible to uncouple muscle loss from weight loss [8, 9] (Figure 1).

**FIGURE 1:**
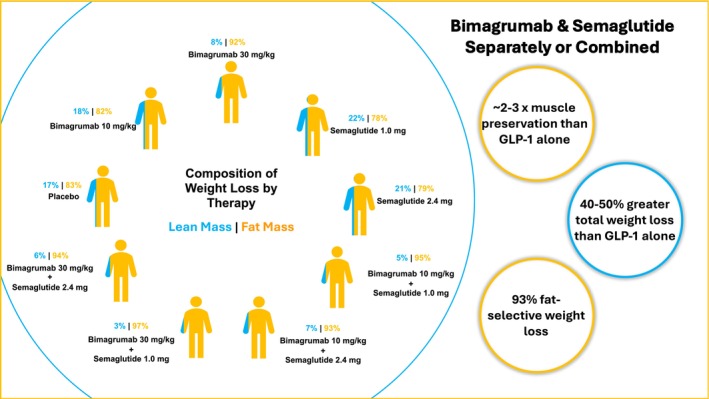
Summary of the composition of weight loss (lean mass and fat mass) for each of the nine, equal cohorts in the BELIEVE trial.

The BELIEVE trial's findings are highly relevant to the field of cachexia and sarcopenia. In sarcopenic obesity, excess adiposity coexists with reduced muscle mass and is associated with worse outcomes [11]. Preservation of muscle is tied to clinical outcomes and is a key determinant of metabolic health, physical function and resilience to stressors [12, 13]. In cardiometabolic disease, including heart failure, skeletal muscle loss is linked to exercise intolerance, reduced quality of life and adverse outcomes [14, 15]. Weight loss strategies that can reduce adipose tissue but preserve skeletal muscle may provide synergistic benefits in these populations.

In comparison to bariatric surgery, currently the most effective weight loss intervention, the BELIEVE trial's results are a sign this therapy could encroach on surgery [16]. Surgical approaches, including the Roux‐en‐Y gastric bypass and sleeve gastrectomy, are associated with total body weight reductions that still comprise of approximately 20%–30% lean mass reduction [17, 18]. Loss of skeletal muscle postbariatric surgery has been associated with reduction in strength and functional capacity. Additionally, bariatric surgeries carry procedural risks, require sustained nutritional support and may present challenges with weight maintenance [17, 19]. In this context, a nonsurgical therapy capable of achieving substantial total weight reduction and preserve or improve muscle mass is favourable.

Although bariatric surgery confers additional metabolic and hormonal benefits beyond weight loss alone, including glycemic control and cardiovascular event risk reduction, these effects are not exclusive to surgery. The SELECT trial demonstrated that semaglutide for weight loss reduces cardiovascular risk, and the BELIEVE trial continued to observe the beneficial effects on glycemic control in those that were on semaglutide [3, 8].

Notable strengths of the BELIEVE trial include its study design being randomized, double‐blind and placebo controlled across multiple treatment arms. The comparison of different monotherapy and combination therapy dose arms limits confounding and increases the interpretability of treatment effects.

Despite these promising signals, there are some limitations. Adverse events were generally consistent with known side effects. Semaglutide predominately had gastrointestinal side effects, and bimagrumab was associated most with muscle spasms [8]. Tolerability of therapy is essential for widespread adoption, as discontinuation of pharmacological weight loss therapies has been associated with regaining weight. Additionally, long‐term consequences of sustained activin receptor inhibition are not fully understood [9]. The BELIEVE trial authors accurately point out that bimagrumab is currently administered intravenously, which in its present form could limit utilization.

Overall, the BELIEVE trial highlights the need to reframe our target in obesity from absolute weight loss to a more specific body composition. The quality of weight loss may prove to be just as important as the magnitude of weight loss. As our tools, including body mass index, fail to distinguish between adipose tissue and lean skeletal muscle, there is a need to better characterize obesity in patients, particularly those with sarcopenia. Afterall, obesity is fundamentally a disease of excess adiposity, not necessarily weight alone. Future investigations must incorporate functional endpoints including muscle strength and exercise capacity and consider frailty as an endpoint.

This strategy is not isolated, as multiple parallel efforts are underway to preserve lean mass, such as with enobosarm, in clinical trials (NCT06282458) [20].

In conclusion, by addressing both incretin‐based and activin receptor signalling pathways, the combination of semaglutide with bimagrumab paves the way for muscle‐preserving pharmacotherapy in weight loss. Whether this approach will ultimately translate into improved patient outcomes remains to be determined, but I believe it is a clear signal of an advancing paradigm shift in treating obesity and its treatment complications.

## Conflicts of Interest

JDA has no conflicts of interests to declare. SDA has received grants and personal fees from Vifor and Abbott Vascular and personal fees for consultancies, trial committee work and/or lectures from Actimed, Amgen, AstraZeneca, Bayer, Boehringer Ingelheim, BioVentrix, Brahms, Cardiac Dimensions, Cardior, Cordio, CVRx, Cytokinetics, Edwards, Farraday Pharmaceuticals, GSK, HeartKinetics, Impulse Dynamics, Novartis, Occlutech, Pfizer, Repairon, Sensible Medical, Servier, Vectorious and V‐Wave. He is the co‐inventor of two patent applications regarding MR‐proANP (DE 102007010834 and DE 102007022367), but he does not personally benefit from the related issued patents. MSK has received fees from Bayer, Boehringer Ingelheim and Novartis.

## Data Availability

Data sharing is not applicable to this article, as no datasets were generated or analysed during the current study.

## References

[1] J. P. H. Wilding , R. L. Batterham , S. Calanna , et al., “Once‐Weekly Semaglutide in Adults With Overweight or Obesity,” New England Journal of Medicine 384, no. 11 (2021): 989–1002. 33567185 10.1056/NEJMoa2032183

[2] D. Rubino , N. Abrahamsson , M. Davies , et al., “Effect of Continued Weekly Subcutaneous Semaglutide vs Placebo on Weight Loss Maintenance in Adults With Overweight or Obesity: The STEP 4 Randomized Clinical Trial,” Journal of the American Medical Association 325, no. 14 (2021): 1414–1425. 33755728 10.1001/jama.2021.3224 PMC7988425

[3] A. M. Lincoff , K. Brown‐Frandsen , H. M. Colhoun , et al., “Semaglutide and Cardiovascular Outcomes in Obesity Without Diabetes,” New England Journal of Medicine 389, no. 24 (2023): 2221–2232. 37952131 10.1056/NEJMoa2307563

[4] S. D. Anker , L. Ji , T. Kindel , et al., “iCARDIO Alliance Global Implementation Guidelines for the Management of Obesity 2025—Focus on Prevention and Treatment of Cardiometabolic Disease,” Global Cardiology 3 (2025): 181–206. 10.1016/j.ajpc.2026.101445 PMC13261179 42291066

[5] S. B. Heymsfield , M. C. Gonzalez , W. Shen , L. Redman , and D. Thomas , “Weight Loss Composition Is One‐Fourth Fat‐Free Mass: A Critical Review and Critique of This Widely Cited Rule,” Obesity Reviews 15, no. 4 (2014): 310–321. 24447775 10.1111/obr.12143 PMC3970209

[6] C. W. le Roux , A. Astrup , K. Fujioka , et al., “3 Years of Liraglutide Versus Placebo for Type 2 Diabetes Risk Reduction and Weight Management in Individuals With Prediabetes: A Randomised, Double‐Blind Trial,” Lancet 389, no. 10077 (2017): 1399–1409. 28237263 10.1016/S0140-6736(17)30069-7

[7] J. R. Lundgren , C. Janus , S. B. K. Jensen , et al., “Healthy Weight Loss Maintenance With Exercise, Liraglutide, or Both Combined,” New England Journal of Medicine 384, no. 18 (2021): 1719–1730. 33951361 10.1056/NEJMoa2028198

[8] S. B. Heymsfield , L. J. Aronne , P. Montgomery , et al., “Bimagrumab Plus Semaglutide Alone or in Combination for the Treatment of Obesity: A Randomized Phase 2 Trial,” Nature Medicine 32, no. 3 (2026): 869–882. 10.1038/s41591-026-04204-0 PMC13004672 41772149

[9] H. Amthor and W. M. Hoogaars , “Interference With Myostatin/ActRIIB Signaling as a Therapeutic Strategy for Duchenne Muscular Dystrophy,” Current Gene Therapy 12, no. 3 (2012): 245–259. 22554312 10.2174/156652312800840577

[10] L. A. Khan , M. Shahzeb Khan , R. Usman Latif , and M. S. Anker , “Cardiac Wasting in Patients With Advanced Cancer: State of the Art Review,” Global Cardiology 3 (2025): 26–36.

[11] C. M. Prado , J. C. Wells , S. R. Smith , B. C. Stephan , and M. Siervo , “Sarcopenic Obesity: A Critical Appraisal of the Current Evidence,” Clinical Nutrition 31, no. 5 (2012): 583–601. 22809635 10.1016/j.clnu.2012.06.010

[12] R. R. Wolfe , “The Underappreciated Role of Muscle in Health and Disease,” American Journal of Clinical Nutrition 84, no. 3 (2006): 475–482. 16960159 10.1093/ajcn/84.3.475

[13] A. J. Cruz‐Jentoft , G. Bahat , J. Bauer , et al., “Sarcopenia: Revised European Consensus on Definition and Diagnosis,” Age and Ageing 48, no. 1 (2019): 16–31. 30312372 10.1093/ageing/afy169 PMC6322506

[14] S. D. Anker , J. E. Morley , and S. von Haehling , “Welcome to the ICD‐10 Code for Sarcopenia,” Journal of Cachexia, Sarcopenia and Muscle 7, no. 5 (2016): 512–514. 27891296 10.1002/jcsm.12147 PMC5114626

[15] S. D. Anker , M. Shahzeb Khan , L. Arshad Khan , et al., “The Muscle Hypothesis of Shortness of Breath in Patients With Cachexia,” Global Cardiology 2 (2024): 223–226.

[16] L. Sjöström , “Review of the Key Results From the Swedish Obese Subjects (SOS) Trial—A Prospective Controlled Intervention Study of Bariatric Surgery,” Journal of Internal Medicine 273, no. 3 (2013): 219–234. 23163728 10.1111/joim.12012

[17] F. Carrasco , K. Papapietro , A. Csendes , et al., “Changes in Resting Energy Expenditure and Body Composition After Weight Loss Following Roux‐en‐Y Gastric Bypass,” Obesity Surgery 17, no. 5 (2007): 608–616. 17658019 10.1007/s11695-007-9117-z

[18] T. B. Chaston and J. B. Dixon , “Factors Associated With Percent Change in Visceral Versus Subcutaneous Abdominal Fat During Weight Loss: Findings From a Systematic Review,” International Journal of Obesity 32, no. 4 (2008): 619–628. 18180786 10.1038/sj.ijo.0803761

[19] S. Stegen , W. Derave , P. Calders , C. van Laethem , and P. Pattyn , “Physical Fitness in Morbidly Obese Patients: Effect of Gastric Bypass Surgery and Exercise Training,” Obesity Surgery 21, no. 1 (2011): 61–70. 19997987 10.1007/s11695-009-0045-y

[20] ClinicalTrials.gov , “Dose‐Finding Study Evaluating Effect on Body Composition of Enobosarm in Patients Taking a GLP‐1 for Chronic Weight Mgmt (QUALITY) (NCT06282458),” https://clinicaltrials.gov/study/NCT06282458.

